# Evaluation of Intereye Corneal Asymmetry in Patients with Keratoconus. A Scheimpflug Imaging Study

**DOI:** 10.1371/journal.pone.0108882

**Published:** 2014-10-08

**Authors:** Lóránt Dienes, Kinga Kránitz, Éva Juhász, Andrea Gyenes, Ágnes Takács, Kata Miháltz, Zoltán Z. Nagy, Illés Kovács

**Affiliations:** 1 Semmelweis University, Department of Ophthalmology, Budapest, Hungary; 2 Karl Landsteiner Institute of Process Optimalization and QM in Cataract Surgery, Vienna, Austria; Save Sight Institute, Australia

## Abstract

**Purpose:**

To assess the correlation between keratoconus severity and intereye asymmetry of pachymetric data and posterior elevation values and to evaluate their combined accuracy in discriminating normal corneas from those with keratoconus.

**Methods:**

This study included 97 patients: 65 subjects with bilateral normal corneas (NC) and 32 with keratoconus (KC). Central corneal thickness (CCT), thinnest corneal thickness (ThCT) and posterior elevation (PE) at the thinnest point of the cornea were measured in both eyes using Scheimpflug imaging. Intereye asymmetry and its correlation with keratoconus severity were calculated for each variable. The area under the receiver operating characteristic curve (AUROC) was used to compare predictive accuracy of different variables for keratoconus.

**Results:**

In normal eyes, intereye differences were significantly lower compared with the keratoconus eyes (p<0.001, for CCT, ThCT and PE). There was a significant exponential correlation between disease severity and intereye asymmetry of steep keratometry (r^2^ = 0.55, p<0.001), CCT (r^2^ = 0.39, p<0.001), ThCT (r^2^ = 0.48, p<0.001) and PE (r^2^ = 0.64, p<0.001). After adjustment for keratoconus severity, asymmetry in thinnest pachymetry proved to be the best parameter to characterize intereye corneal asymmetry in keratoconus. This variable had high accuracy and significantly better discriminating ability (AUROC: 0.99) for KC than posterior elevation (AUROC: 0.96), ThCT (AUROC: 0.94) or CCT (AUROC: 0.92) alone.

**Conclusions:**

There is an increased intereye asymmetry in keratometry, pachymetry and posterior corneal elevation values in keratoconic patients compared to subjects with normal corneas. Keratoconus patients with more severe disease are also more asymmetric in their disease status which should be taken into account during clinical care.

## Introduction

Keratoconus is a progressive, bilateral corneal ectatic disease [1] with initial unilateral presentation between 0.5%–4.5% [2]–[6]. Previous studies have shown that patients with an initially unilateral form commonly develop signs of keratoconus in the other eye as well, with a reported frequency of 50% in clinically normal fellow eyes within 16 years [4], [7]. These results suggest that the majority of patients have bilateral disease but its presentation is asymmetric between the two eyes [8]–[10]. The asymmetry in keratoconic patients in terms of clinical signs, corneal curvature, and topographic indices have already been published and was used as a diagnostic criterion of keratoconus [8], [9]. The Pentacam Comprehensive Eye Scanner (Oculus Optikgerate GmbH, Wetzlar, Germany) uses a rotating Scheimpflug camera and represents a sensitive device for detecting subtle changes of the corneal surface and allows detailed qualitative and quantitative analysis of the corneal shape. In keratoconus, the most specific changes in curvature are steepening and protrusion of the cornea in parallel with significant thinning of the corneal stroma, which usually occurs inferior to the visual axis. The Pentacam Scheimpflug camera asseses the curvature and elevation of the anterior and posterior corneal surface as well as pachymetry with high reproducibility and repeatability [11], [12]. Several studies have proved high accuracy of posterior elevation measurements in detecting keratoconus [13]–[17] and some reported pachymetry as a sensitive parameter to detect progressive changes in keratoconus [18]–[24]. In addition, relational thickness profile was found to be superior to single-point pachymetric data in discriminating normal corneas from those with keratoconus [25], [26]. Recently, corneal pachymetry and posterior elevation maps (corneal tomographic maps) are used frequently in clinical practice for evaluating both refractive surgery candidates and keratoconic patients [27], [28]. One previous study reported significantly increased intereye variability of pachymetric data and posterior elevation values in keratoconic eyes compared to normals [29], however there are no data on the effect of keratoconus severity on intereye asymmetry. The purpose of this study was to assess the correlation between keratoconus severity and intereye asymmetry of pachymetric data and posterior elevation values and to evaluate their combined accuracy in discriminating normal corneas from those with keratoconus.

## Methods

This study evaluated patients with mild to moderate keratoconus (KC group) and eyes of refractive surgery candidates (control group). Both eyes of each patient in both groups were used. Eyes with severe keratoconus were excluded because of difficulties in topographic map acquisition and potential stromal haze or scar formation, which can alter the optical transparency of the cornea and thus Scheimpflug imaging. Severe keratoconus was defined as having axial topographic pattern consistent with keratoconus, positive slit lamp findings, and an average corneal power higher than 56 D or dense/opaque corneal scarring according to the Keratoconus Severity Score criteria [30]. Both eyes of each patient had a complete ophthalmologic evaluation including slit lamp biomicroscopy, keratometry, retinoscopy, slit lamp indirect ophthalmoscopy, and Placido disk–based videokeratography (TOMEY TMS-4 corneal topographer; TOMEY Corp., Nagoya, Japan). Diagnosis was based on classic corneal biomicroscopic and topographic findings in accordance with the criteria of Rabinowitz et al. [1]. Inclusion criteria for the control group included a refractive error less than 5.00 diopters (D) sphere and astigmatism less than 3.00 D. None of the control patients had a history of previous ocular disease, surgery or trauma. Rigid contact lenses were not worn for 4 weeks and soft contact lenses for at least 1 week before assessment in either groups. Patients were asked whether they rubbed their eyes or experienced previous ocular trauma. The study was conducted in compliance with the Declaration of Helsinki, applicable national and local requirements regarding the ethics committee and institutional review boards. Ethical approval was obtained from the Institutional Review Board (Semmelweis University Regional and Institutional Committee of Sciences and Research Ethics). A written informed consent was obtained before the examination from each patient.

### Scheimpflug assessment

All eyes were examined with the Pentacam HR Scheimpflug camera, used by three trained examiners without application of dilating or anaesthetic eye drops or previous tonometry. The readings were taken as recommended in the instruction manual. The measurement results were checked under the quality specification (QS) window, only the correct measurements (‘QS’ reads OK) were accepted; if the comments were marked yellow or red, the examination was repeated. In all cases one reading taken from an eye was saved and processed for further statistical analyses. For local posterior elevation measurements, the reference surface was set to best fit sphere (BFS) with fixed 8- mm-diameter settings. Keratometry at the steep (K_s_) and flat (K_f_) meridians, central corneal thickness (CCT), pachymetry at the thinnest point (ThCT) and posterior elevation at the thinnest point of the cornea (PE) were measured in both eyes. Intereye asymmetry of pachymetry and elevation data was determined by subtracting the lower value from the higher value for each variable. The better and worse eyes were designated for each keratoconus patient based on each variable (i.e. the worse eye is with higher K_s_, K_f_, PE and lower CCT and ThCT).

### Statistical analysis

Statistical analysis was performed with SPSS software (version 15.0, SPSS, Inc.). The Shapiro-Wilk W test was used to confirm normal distribution of the variables. Paired samples t-test was used to compare means between eyes of the same subject (within-subject variance). Linear regression was used to test significant correlation between parameters of the two eyes of the same subject (within-subject correlation). The repeated measures analysis of variance test (ANOVA) was used to analyze the differences between group means and their associated procedures (within-group and between-group variances). This test allows to compare within-subject parameters (better eye vs. worse eye) in the two study groups by taking into account between-eye correlations by treating data from eyes of patients in statistical analysis as repeated measures. Correlation between keratoconus severity and intereye asymmetry was tested using linear and non-linear regression analysis in each group. In this study keratoconus severity was assessed by corneal thickness values as it was suggested previously [25]. Receiver operator characteristic curves (ROCs) with covariate adjustment were used to compare discriminating ability of posterior elevation and pachymetry data after adjustment for the correlation between keratoconus severity and between-eye asymmetry. In ROC analysis, covariate adjustment is recommended when the accuracy of the test result is dependent on patient characteristic, similarly as adjusting for confounders in multivariable regression. In all analyses, a P value less than 0.05 was considered as statistically significant.

## Results

The keratoconus group comprised 64 eyes of 32 patients (15 men, 17 women) with a mean age of 36.98±12.34 years. The control group comprised 130 eyes of 65 patients (29 men, 36 women) with a mean age of 39.95±15.44 years. There were no statistically significant differences between the keratoconus and the control groups in age or sex distribution (p>0.05). Table 1 summarizes mean and standard deviation values of topographic, posterior elevation and pacyhmetry parameters in the two groups. We have found no significant correlation between self-reported eye rubbing or ocular trauma and the presence of keratoconus in a given eye (p>0.05).

**Table 1:** Mean ± SD value for each parameter in the Keratoconus and Control Groups.

*Parameter*	*Keratoconus Group*		*Control Group*		*p*	
	*Better eye*	*Worse eye*	*Right eye*	*Left eye*	*Between* *eye* †	*Between group* ††
***K_f_ (D)*** *	44.90±3.09	47.42±4.58	42.69±1.62	42.92±1.57	*<0.001/*>0.05	*<0.001*
***K_s_ (D)*** *	46.84±4.23	51.33±5.56	43.92±1.67	44.32±1.93	*<0.001/*>0.05	*<0.001*
***CCT (µm)^#^***	493.73±26.04	463.60±33.53	554.62±26.98	557.31±27.18	*<0.001/*>0.05	*<0.001*
***ThCT (µm)^#^***	493.53±47.07	453.83±47.59	546.33±30.91	551.82±28.48	*<0.001/*>0.05	*<0.001*
***PE (µm)*** *	32.60±29.51	68.00±51.24	6.71±6.42	5.38±6.06	*<0.001/*>0.05	*<0.001*

*Worse eye is the eye with the highest value and ^#^Worse eye is the eye with the lowest value.

† *Worse eye vs. better eye in the Keratoconus Group/Right eye vs. left eye in the Control Group; Student’s t-test on dependent samples.*

†† *Keratoconus vs. Control groups; Student’s t-test on independent samples.*

*PE: posterior elevation; CCT: central corneal thickness; ThCT: thinnest corneal thickness.*

There was a statistically significant difference in keratometric, CCT, ThCT and PE values between *worse eye* and *better eye* in the keratoconus group (Table 1). In contrast, there was no significant difference in these parameters between the *right eye* and the *left eye* of controls (Table 1). We found significantly higher values of posterior elevation, flat and steep keratometry (p<0.001, for all of the parameters) and significantly decreased central and thinnest pachymetry values in the keratoconus group compared to controls (p<0.001, for both parameters, Table 1). As Table 2 presents, mean intereye difference was significantly higher for all of the variables when comparing keratoconus eyes with normal eyes (p<0.001).

**Table 2:** Mean intereye asymmetry of each parameter in the keratoconus and in the control groups.

*Parameter*	*Keratoconus Group*		*Control Group*		*p*
	*Mean intereye asymmetry*	*Range*	*Mean intereye asymmetry*	*Range*	
***K_f_ (D)***	2.70±3.57	0.3–13.8	0.37±0.39	0–1.5	*<0.001*
***K_s_ (D)***	4.37±5.14	0.1–20.2	0.43±0.44	0–2.3	*<0.001*
***PE (µm)***	35.4±37.31	0–161	3.13±3.71	0–21	*<0.001*
***ThCT (µm)***	39.70±36.42	0–136	6.57±5.30	0–18	*<0.001*
***CCT (µm)***	30.13±35.80	3–113	5.59±4.90	0–18	*<0.001*

*p: Student’s t-test for independent samples.*

*PE: posterior elevation; CCT: central corneal thickness; ThCT: thinnest corneal thickness.*

**Figure 1:**
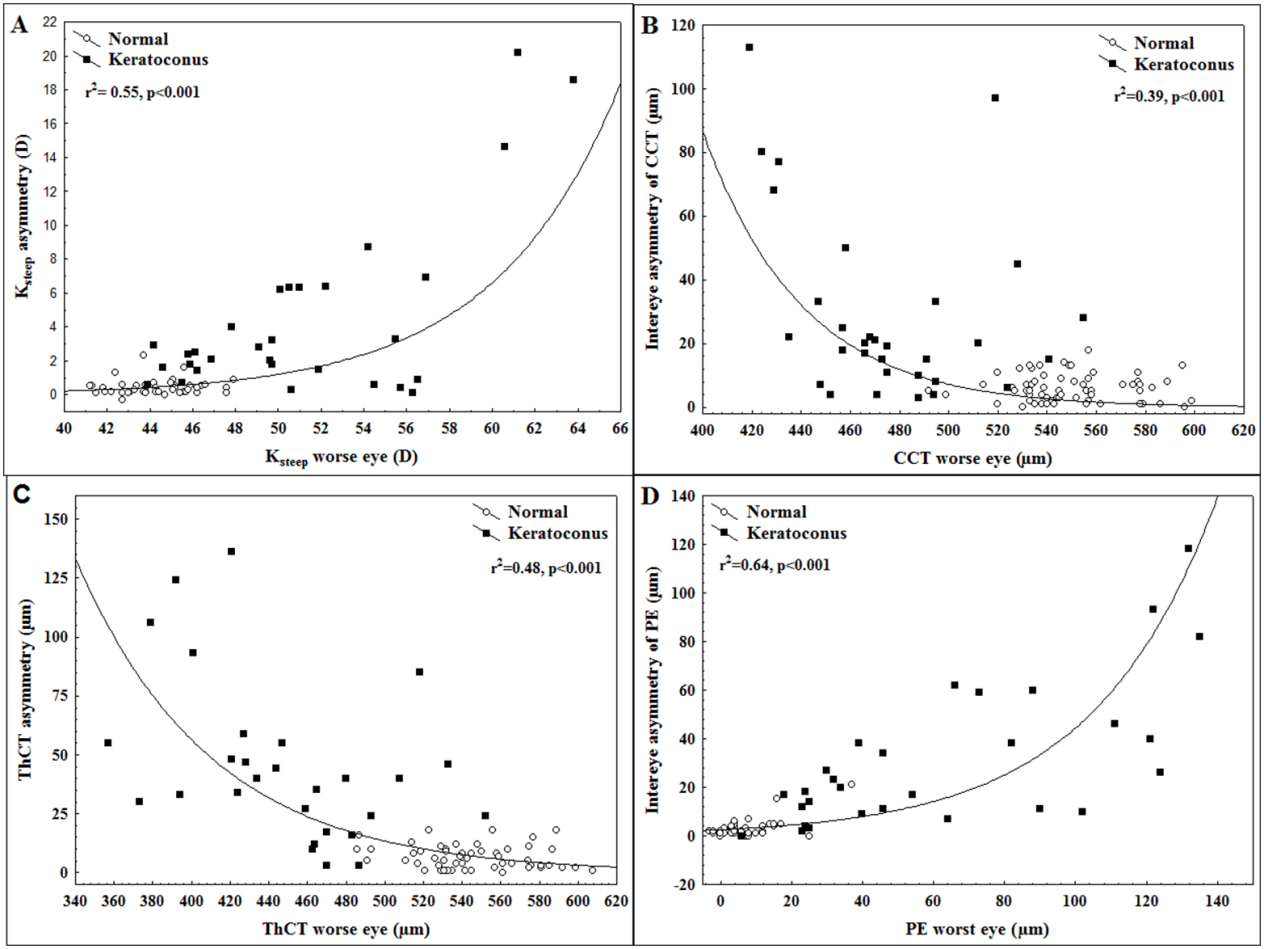
The relationship between keratoconus severity and intereye asymmetry. Exponential regression curve fit to data of steep keratometry (K_steep_; **1A**), central corneal thickness (CCT; **1B**), thinnest corneal thickness (ThCT; **1C**) and posterior elevation (PE; **1D**) from the two study groups.

Correlation analysis showed significant correlation between data from the *worse eye* and data from the *better eye* in the keratoconus group (p<0.001, Table 3). Data from the *right eye* and data from the *left eye* in the control group also showed strong correlation (p<0.001, Table 3). The difference between correlation coefficients was significant for each variable (Table 3). Intereye asymmetry of pachymetry significantly correlated with decreasing thinnest pachymetry (r = −0.40; p = 0.03) or central pachymetry (r = −0.72; p = 0.002) in the keratoconus group but not in the control group (p>0.05). Similarly, correlation was found between intereye asymmetry of PE and increasing posterior elevation (r = 0.82; p<0.001) in the keratoconus group but not in the control group (p>0.05). The relationship between intereye asymmetry and keratoconus severity could best be described by an exponential regression model across the two groups with an r value of 0.74 for steep keratometry (r^2^ = 0.55, p<0.001; Figure 1A), with an r value of 0.62 for CCT (r^2^ = 0.39, p<0.001; Figure 1B), an r value of 0.69 for ThCT (r^2^ = 0.48, p<0.001; Figure 1C) and an r value of 0.80 for PE (r^2^ = 0.64, p<0.001; Figure 1D).

**Table 3:** Correlations between data from the two eyes in the keratoconus group, and in the control group.

*Parameter*	*Keratoconus group*	*Control group*	*p*
***Posterior elevation (µm)***	*r = 0.70; p<0.001*	*r = 0.87; p<0.001*	*0.003*
***Thinnest corneal thickness (µm)***	*r = 0.70; p<0.001*	*r = 0.98; p<0.001*	*<0.001*
***Central corneal thickness (µm)***	*r = 0.68; p<0.001*	*r = 0.98; p<0.001*	*<0.001*

*p: difference between r values of the two groups.*

**Figure 2:**
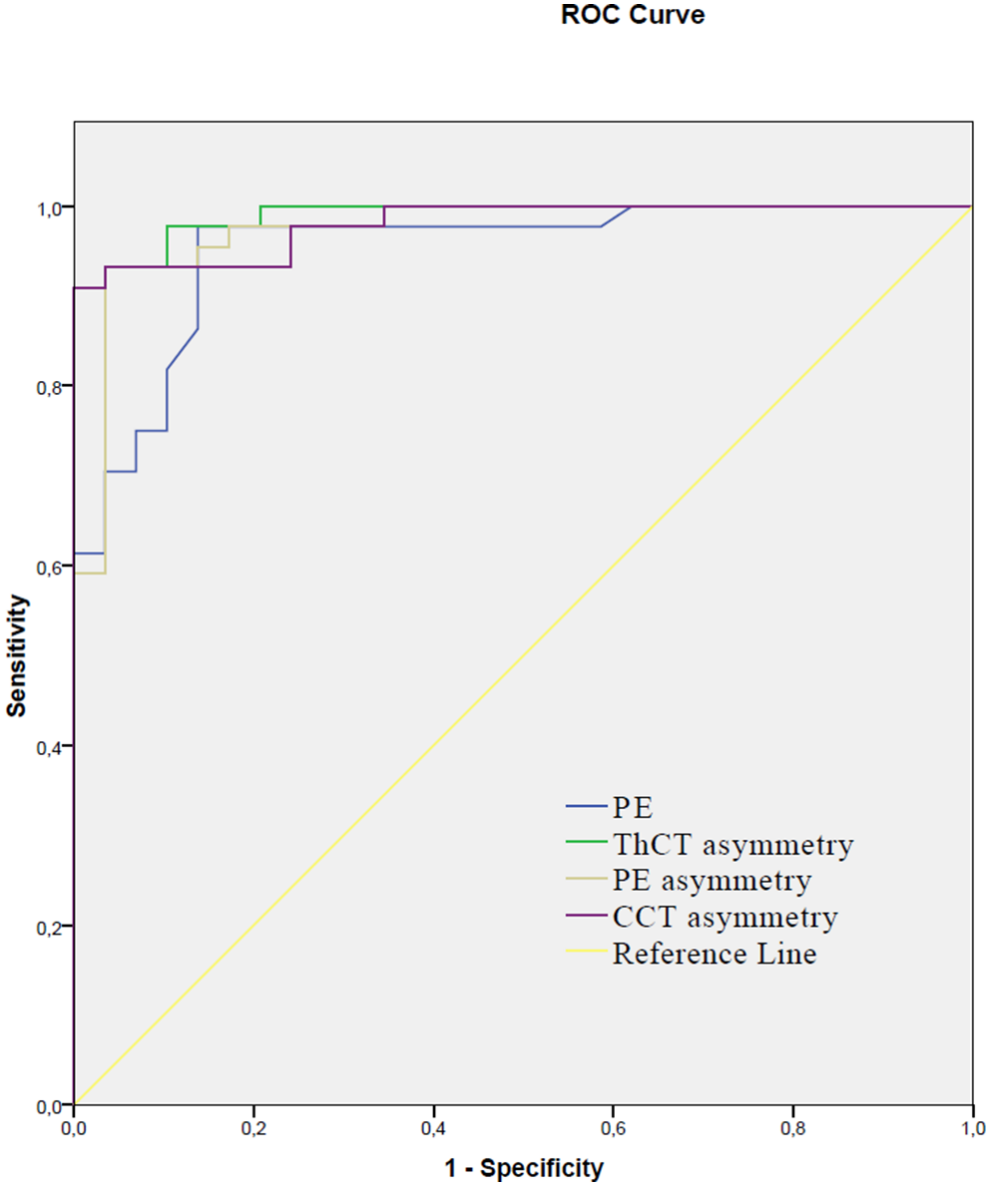
Receiver operator characteristic curves to plot discriminating ability of the different parameters for keratoconus. See corresponding AUROC values for posterior elevation (PE), asymmetry in central corneal thickness (CCT), posterior elevation (PE) and thinnest corneal thickness (ThCT) in Table 4.

To identify the best parameter to characterize intereye corneal asymmetry in keratoconus, receiver operator characteristic curves with adjustment for keratoconus severity was used. This ROC analysis showed, that asymmetry in thinnest pachymetry had the highest accuracy (AUROC: 0.99) and significantly better discriminating ability for keratoconus than posterior elevation (AUROC: 0.96), ThCT (AUROC: 0.94) or CCT had (AUROC: 0.92; pairwise comparison p<0.05, Figure 2, Table 4).

**Table 4:** Area under the ROC curve values with 95% confidence limits and pairwise comparisons of different variables for keratoconus vs. normals.

*Parameter*	*AUROC*	*95% CL*	*Cut off*	*Sensitivity*	*Specificity*	*p*	*p* †
***PE***	0.96	0.90–0.99	17.5	97	91	*<0.001*	-
***CCT***	0.92	0.85–0.97	513	91	93	*<0.001*	0.37
***ThCT***	0.94	0.87–0.97	509	93	89	*<0.001*	0.24
***PE asymmetry***	0.97	0.90–0.99	7	97	93	*<0.001*	0.33
***CCT asymmetry***	0.98	0.94–0.99	10	97	94	*<0.001*	0.18
***ThCT asymmetry***	0.99	0.97–1.00	12	98	95	*<0.001*	*0.03*

† *Pairwise comparison to AUROC value of PE.*

*PE: posterior elevation; CCT: central corneal thickness; ThCT: thinnest corneal thickness.*

## Discussion

We found significantly increased intereye difference in posterior elevation and pachymetry values in keratoconus patients compared to normals, confirming previous reports [29]. We also proved, that there is a strong correlation between the two eyes of the same subject (within-subject correlation) both in healthy persons and those with keratoconus in posterior elevation and pachymetry values. In terms of these parameters the finding in one eye predicts the finding in the fellow eye almost perfectly in healthy persons and moderately in keratoconus patients. The decreased correlation between values measured in the two eyes of the same subject with keratoconus is a consequence of the asymmetrical nature of this disease.

In this study there was no significant difference in posterior elevation and pachymetry parameters comparing right eyes to left eyes (p>0.05 for all of the variables) in each group due to the lack of side predilection in keratoconus. In contrast, after categorizing eyes into *“worse eye”* and *“better eye”* we found significant intereye differences for all of the variables in the keratoconus group. The strong correlation of data from the two eyes (between-eye symmetry) together with the small variability of data in the group (between-subject similarity) are characteristic features of the normal group. In the keratoconus group, there were decreased between-eye correlation and increased variability of data as a result of decrease in “between-eye symmetry” and “between-subject similarity” which changes are characteristic features of this progressive, asymmetric disease. An important finding of this study is that keratoconus severity was significantly correlated with intereye asymmetry of keratometric, pachymetric and elevation values with a smooth transition as it was demonstrated with good fit of exponential curves to data. Keratoconus is a progressive disorder ultimately affecting both eyes, although initially only one eye may be affected. It is also known, that atypical, asymmetric topography pattern in normal fellow eyes is associated with higher risk for the development of keratoconus [7]. Previous studies introduced different indices and proposed cut-off values to identify different stages of KC, however, for any quantitative variable there is a significant overlap between KC suspect and normals resulting in lower sensitivity and specificity in detecting mild corneal ectasia compared to discriminating normal corneas from keratoconus. Progression of a chronic disease, like keratoconus is often depicted in three states: normal, preclinical phase and clinical phase [31] and the screening of the asymptomatic preclinical phase is usually much more difficult than of the symptomatic clinical phase. A clear understanding of progression from the preclinical phase to the clinical phase is therefore important for keratoconus screening. One previous study reported significantly increased keratometric, topometric and elevation parameters in normal fellow eyes of unilateral keratoconus patients compared to normals [32]. According to their results, keratometric asymmetry, topometric indices and anterior/posterior elevation difference may be useful in detecting the earliest form of subclinical keratoconus. In this study, we found exponential correlation of corneal asymmetry with pachymetric severity from healthy to keratoconus. After this correlation with intereye asymmetry of ThCT was taken into account by the ROC analysis, we found significantly better discriminating ability for keratoconus as using posterior elevation or pachymetry data alone (Figure 2, Table 4). In a previous study, Ambrosio et al. described high AUROC values for ThCT and CCT for discriminating keratoconus (0.955 and 0.909 respectively) [25], however pachymetric asymmetry was not considered in these analyses. In our pacyhmetry adjusted analysis ThCT asymmetry had significantly better discriminating ability for keratoconus (AUROC: 0.99) than posterior elevation had (AUROC: 0.96, Table 4). The pachymetry adjusted ThCT asymmetry utilized all the three significant pachymetric characteristics of keratoconus (lower ThCT, higher variance of ThCT and correlation of ThCT with asymmetry of ThCT) simultaneously for keratoconus prediction. This method showed the best accuracy in discriminating keratoconus cases from normals comparing ROC curves (Figure 2) with high sensitivity and specificity (98% and 95%, respectively). All these findings suggest that simultaneous analysis of both intra- and intereye asymmetry could be utilized to further improve the diagnostic accuracy of keratoconus. When plotted as a function of the corresponding minimum pachymetry, intereye ThCT asymmetry tended to exponentially increase with decreasing thinnest corneal thickness (Figure 1). One clinical relevance of this finding is that increased pachymetric asymmetry can be a warning sign for the presence of keratoconus in subjects with pachymetric values in the subnormal or normal range, often posing diagnostic problems [33]. According to results of the ROC analysis, asymmetry in corneal pachymetry has good accuracy in predicting keratoconus, when its correlation with disease severity is also taken into account. When controlling for corneal thickness, values of intereye pachymetric asymmetry beyond 10 µm for CCT and 12 µm for ThCT should warn the clinician for a significantly increased risk for the presence of corneal ectasia. These subjects should be processed for further screening for an ectatic disorder and should be assigned for control measurements to detect progressive ectasia. When controlling for the effect of disease severity, the optimal cut-off point for posterior elevation asymmetry was 7 µm and showed 97% sensitivity and 93% specificity in predicting keratoconus. Although these results show, that increased corneal asymmetry predicts keratoconus with good accuracy, the diagnosis of mild cases remains challenging and further studies are needed focusing on simultaneous analysis of within-eye and between-eye asymmetry.

As a conclusion, in this study we have shown that for corneal topography, pachymetry and elevation outcomes, the degree of intereye asymmetry is associated with disease severity. One might conclude from these results that as keratoconus patients proceed through the disease and becoming more severe, more pronounced intereye asymmetry also occurs. In a previous study analysing clinical outcomes of keratoconus, the degree of asymmetry in keratometry, high contrast, best corrected visual acuity, spherical equivalent, and corneal scarring was related to disease severity [34]. According to our results the relation between intereye asymmetry and severity is pronounced in outcomes relating to local corneal changes measured at the apex of the cone. We found exponential correlation of corneal asymmetry in terms of corneal thickness and posterior elevation with pachymetric severity from healthy to keratoconus. This is an important finding as thinnest corneal thickness is directly related to the clinical care of these patients i.e. the application of corneal crosslinking therapy. Increasing pachymetric asymmetry could be thus considered as a warning sign for disease progression and as therapy indication. In our opinion, the fact that all correlations in this study were in the same direction supports the assumption that disease asymmetry and severity are considerably related in keratoconus. However, further studies are recommended as this relation would be better described when longitudinal data were analyzed. Our future analyses will examine whether the progression of keratoconus proceeds in an asymmetric trend or whether the asymmetry observed at baseline in these patients is simply preserved.
